# Extraordinary Case of Somnambulism

**Published:** 1891-08

**Authors:** 


### EXTRAORDINARY CASE OF SOMNAMBULISM.

The *Daily Telegraph's* Paris correspondent says :—An extraordinary case of somnambulism is reported from one of the rural districts. According to the accounts which have reached Paris, the patient is a young man whose legs have been completely paralyzed for some time. In his usual health, he is unable to move without the help of crutches, but when the fit is on him he can walk long distances without the slightest assistance. A few nights ago he got up and started for a neighboring village, followed by some of his relatives, who never lose sight of him when he is in this condition. He arrived without misadventure at the house of a friend, knocked at the door, and asked for refreshment. After having rested for a few moments he returned home, and, as it was still very early in the morning, he sat down on a bench and waited until the people came out of their houses. He then went to bed, and awoke a few hours afterwards without feeling the least fatigue, though he had walked more than ten miles, nor had he the slightest remembrance of the expedition which he had undertaken. The case is said to be exciting the utmost interest throughout the Department, and to be the subject of universal discussion.

